# Ureter metastatic castration-resistant prostate cancer: a case report

**DOI:** 10.1186/s13256-017-1379-z

**Published:** 2017-09-06

**Authors:** Sohgo Tsutsumi, Takashi Kawahara, Yusuke Hattori, Taku Mochizuki, Jun-ichi Teranishi, Yasuhide Miyoshi, Sawako Chiba, Hiroji Uemura

**Affiliations:** 1 Departments of Urology and Renal Transplantation, Yokohama City University Medical Center, Yokohama, Japan; 2 Department of Diagnostic Pathology, Yokohama City University Medical Center, Yokohama, Japan

**Keywords:** Skene’s gland adenocarcinoma, Skene’s gland cancer, Female PSA

## Abstract

**Background:**

In most cases, prostate cancer metastasizes to the lymph nodes, bone, and liver. In very rare cases, it metastasizes to the ureter. Due to the difficulty in making a preoperative diagnosis, ureteral metastasis from prostate cancer is typically diagnosed after nephroureterectomy.

**Case presentation:**

A 77-year-old Asian Japanese man with right hydronephrosis and hydroureter was referred to our hospital to undergo further examination due to the suspicion of ureteral cancer. He had been diagnosed 2 years previously with prostate cancer with a Gleason score of 4+5=9. He received radiotherapy and androgen deprivation therapy. A nephroureterectomy was performed for suspected right ureteral cancer. On the basis of a histopathological examination, poorly differentiated adenocarcinoma was suspected, and the tumor cells were positive for prostate-specific antigen immunohistochemically.

**Conclusions:**

We herein report a rare case of ureteral metastasis in castration resistant prostate cancer.

## Background

In most cases, prostate cancer metastasizes to the lymph nodes, bone, and liver. In very rare cases, it metastasizes to the ureter [1]. Due to the difficulty in obtaining a preoperative diagnosis, ureteral metastasis from prostate cancer is typically diagnosed on the basis of examination of nephron specimens obtained at ureterectomy. We report an extremely rare case involving a patient with ureteral metastasis from prostate cancer.

## Case presentation

**Fig. 1:**
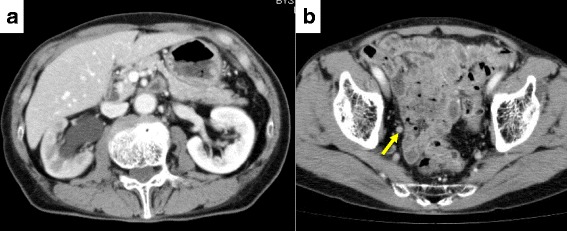
Computed tomographic findings of (**a**) hydronephrosis and (**b**) the patient’s tumor (*arrow*)

**Fig. 2:**
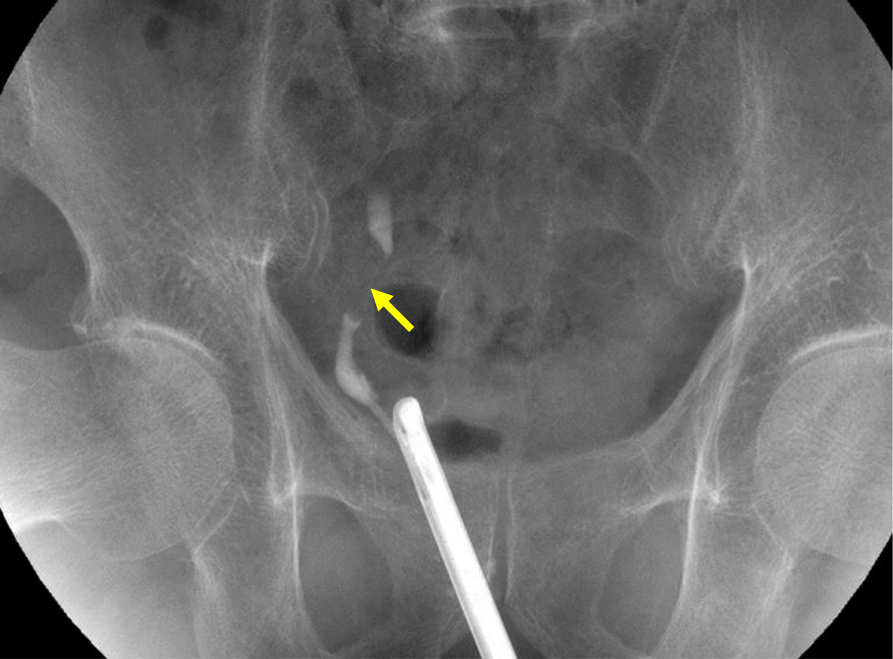
Retrograde pyelonephrography. Stenosis was observed in the lower ureter (*arrow*)

A 77-year-old Asian Japanese man with suspected ureteral cancer was referred to our hospital to undergo further examination for right hydronephrosis and hydroureter in September 2016. He had undergone resection for cecal cancer and had been diagnosed with prostate cancer 2 years previously with a Gleason score of 4+5=9. He received radiotherapy with androgen deprivation therapy (ADT) for cT2N0M0 prostate cancer. The patient’s initial prostate-specific antigen (PSA) level was 14.66 ng/ml in 2014. Although the initial response was good, his PSA level increased to 0.417 ng/ml with continuous ADT (testosterone 0.30 ng/ml) from a nadir of 0.006 ng/ml in 2016. The results of a laboratory analysis were almost within normal limits, with the exception of a slightly elevated creatinine level (1.31 mg/dl) and a slightly decreased hemoglobin level (12.6 ng/dl). Urinalysis revealed no abnormalities, and urine cytology showed no atypical cells. Enhanced computed tomography showed right hydronephrosis and hydroureter from a lower ureteral mass with enhancement (Fig. 1). Retrograde pyelography showed complete obstruction of the right ureter (Fig. 2). Although the patient had castration-resistant prostate cancer (CRPC), the median overall survival was around 3 years at our institute, and the patient requested to undergo curative surgery for his ureteral tumor.

On the basis of these findings, we suspected right ureteral cancer and performed a nephroureterectomy in December 2016. The ureter showed strong adhesion to the peritoneum and was partially removed with the peritoneum. The resected specimen measured 16 cm × 2 cm in size, and the tumor was observed to have extended to the surface of the resected tissue.

**Fig. 3:**
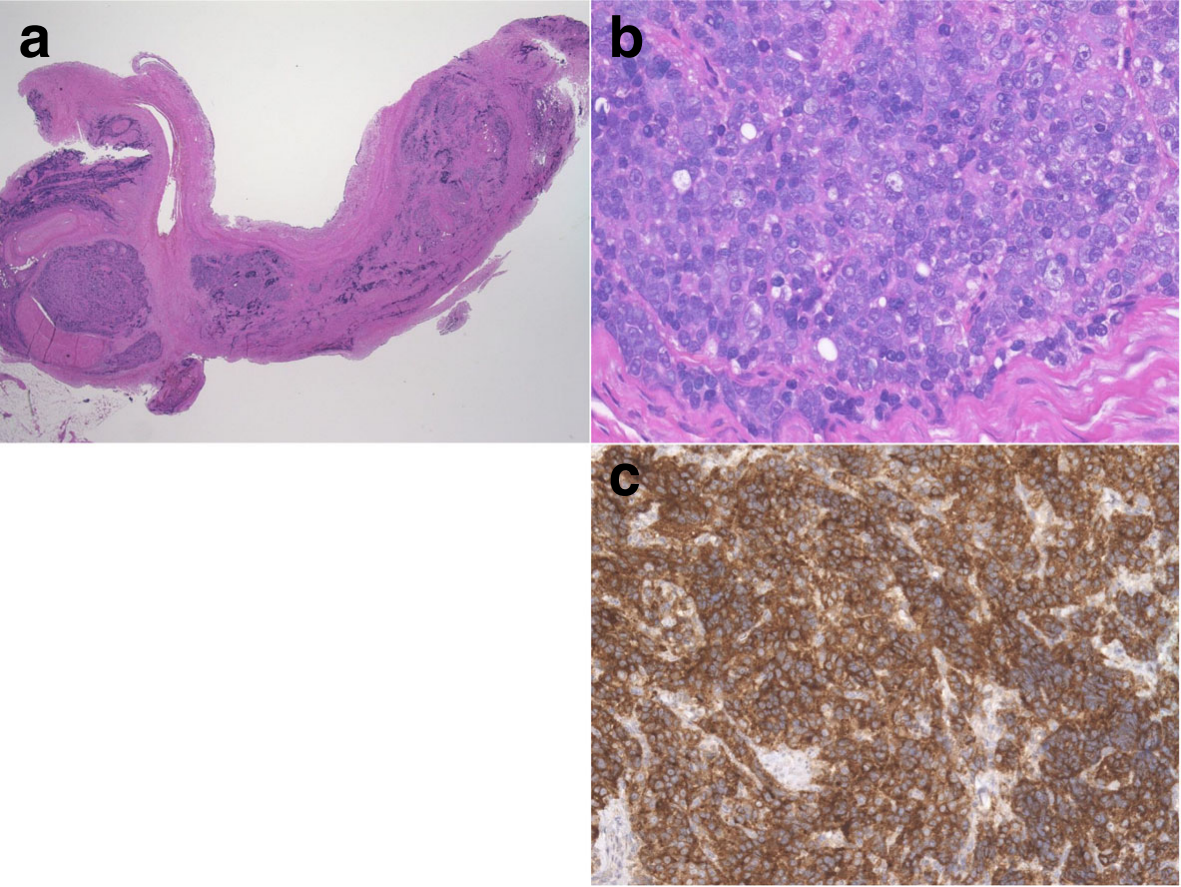
Pathological images. **a** Ureteral epithelium was intact. Solid metastatic nest in intra- and extraureteral wall (hematoxylin and eosin stain, original magnification ×12.5). **b** Tumor showed a little glandular differentiation (hematoxylin and eosin stain, original magnification ×400). **c** Tumor cells stained positive for prostate-specific antigen (original magnification ×200)

Histologically, the ureteral epithelium was normal. In the intra- and extraureteral tissue, tumor cells that had enlarged round nuclei with visible nucleoli were proliferated with a solid pattern. The tumor showed a little glandular differentiation. Poorly differentiated adenocarcinoma was suspected. The result of immunochemical staining for PSA was positive (Fig. 3). On the basis of these findings, the patient was diagnosed with ureteral metastasis from prostate cancer. The patient continues to receive therapy for CRPC.

## Discussion

Disibio *et al*. noted that prostate cancer was likely to metastasize to the lymph nodes (26.2%), bone (19.7%), lung (12.8%), and liver (7.8%) [1]. Ureteral metastasis from prostate cancer is a very rare disease, with only 44 reported cases [2]. It is hypothesized that ureteral metastasis is rare in patients with prostate cancer because the lymph stream is not connected around the ureter [3]. Most patients with ureteral metastasis have primary breast or gastric cancer, whereas some have colon or cervical cancer [4, 5].

Metastatic ureteral tumors are difficult to differentiate from ureteral urothelial carcinoma. Presman *et al*. reported that metastatic ureteral tumors were diagnosed because (1) tumor cells were confirmed in the lymph nodes and vessels around the ureter, or (2) the same cancer cells as the original cancer tissue were detected in the ureteral wall without direct invasion [6]. Metastatic ureteral tumors tend not to adhere to the mucosa, owing to a poor lymphoid and visceral network [6]. For these reasons, pain due to ureteral obstruction is the chief complaint of patients with this type of tumor. In most cases, the tumor is found in the submucosa, and urine cytology is not useful in making the diagnosis. Given recent developments with ureteroscopy, a ureteroscopic biopsy might be useful. However, because of the small sample size, the pathological diagnosis is sometimes difficult.

On one hand, it is hypothesized that the identification of ureteral obstruction and hydronephrosis may be used to make an early diagnosis. On the other hand, patients with benign prostatic hyperplasia and ureteral stones may also show these symptoms. In patients with prostate cancer, ureteral obstruction usually originates from direct invasion from an enlarged tumor around the ureterovesicular junction [7]. In most cases, direct invasion is observed in the bilateral ureterovesicular junction. In our patient, the ureteral obstruction was unilateral and was outside the ureterovesicular junction. In such cases, a ureteroscopic biopsy might be used to make a preoperative diagnosis. However, this strategy is not superior to retrograde pyelography. Fortunately, CRPC treatment does not require favorable renal function, even when cytotoxic treatments are used, including docetaxel and cabazitaxel, so radical nephroureterectomy is sometimes performed.

## Conclusions

We herein report a rare case of ureteral metastasis in castration resistant prostate cancer.
